# Changes in serum albumin and liver enzymes following three different types of bariatric surgery: six-month follow-up. A retrospective cohort study

**DOI:** 10.1590/1516-3180.2021.00065.R1.1504221

**Published:** 2021-10-11

**Authors:** Mohadeseh Hassan Zadeh, Negar Zamaninour, Hastimansooreh Ansar, Ali Kabir, Abdolreza Pazouki, Gholamreza Mohammadi Farsani

**Affiliations:** I Undergraduate Student, Department of Clinical Nutrition, School of Nutritional Sciences and Dietetics, Tehran University of Medical Sciences (TUMS), Tehran, Iran.; II PhD. Nutritionist, Minimally Invasive Surgery Research Center, Iran University of Medical Sciences, Tehran, Iran.; III PhD. Nutritionist, Minimally Invasive Surgery Research Center, Iran University of Medical Sciences, Tehran, Iran.; IV MD, MPH, PhD. Associate Professor, Minimally Invasive Surgery Research Center, Iran University of Medical Sciences, Tehran, Iran.; V MD. Associate Professor of Surgery, Minimally Invasive Surgery Research Center, Iran University of Medical Sciences, Tehran, Iran; and Associate Professor, Center of Excellence of European Branch of International Federation for Surgery of Obesity, Tehran, Iran.; VI MD, MPH, PhD. Assistant Professor, Department of Clinical Nutrition, School of Nutritional Sciences and Dietetics, Tehran University of Medical Sciences (TUMS), Tehran, Iran; and Assistant Professor, Minimally Invasive Surgery Research Center, Iran University of Medical Sciences, Tehran, Iran.

**Keywords:** Bariatric surgery, Metabolic surgery, Transaminases, Serum albumin, Body composition, Roux-en-Y gastric bypass, One-anastomosis gastric bypass, Sleeve gastrectomy

## Abstract

**BACKGROUND:**

Few reports have examined the effects of Roux-en-Y gastric bypass (RYGB), one-anastomosis gastric bypass (OAGB) and sleeve gastrectomy (SG) on changes to serum albumin (Alb) and liver enzyme levels.

**OBJECTIVE:**

To compare short-term post-surgery changes in serum Alb, aspartate aminotransferase (AST), alanine aminotransferase (ALT) and alkaline phosphatase (ALKP) levels. Body composition changes were also measured and compared among three groups.

**DESIGN AND SETTING:**

Retrospective cohort study conducted in Tehran, Iran.

**METHODS:**

151 OAGB, RYGB and SG patients referred to the obesity clinic of Hazrat-e Rasool General Hospital, Tehran, Iran, were evaluated. Physical characteristics and biochemical parameters were measured pre-surgery and then after three and six months.

**RESULTS:**

Through repeated measurements to determine intragroup changes, significant changes in serum AST (P = 0.003) and ALT (P < 0.001) were observed in follow-ups. However, Alb levels did not change (P = 0.413). Body fat, fat-free mass and muscle mass decreased significantly in each group (P < 0.05). In a univariate general linear model for determining intergroup changes, SG showed greater decreases in ALT and AST at three and six months (P < 0.05) and in ALKP at six months (P = 0.037), compared with OAGB. There were no significant differences in Alb levels. Also, RYGB had a greater effect on reducing fat percentage (three months, P = 0.011; six months, P = 0.059) and fat mass (three months, P = 0.042) than OAGB.

**CONCLUSION:**

SG and RYGB may be superior to OAGB in reducing liver enzymes and body fat, respectively. However, Alb levels showed no significant differences.

## INTRODUCTION

The worldwide prevalence of obesity has tripled in the past four decades,^1^ which may have led to higher incidence of some major health problems, such as type 2 diabetes (T2DM), high blood pressure, cardiovascular disease (CVD), degenerative arthritis and sleep apnea.^2^ Trends have also shown that non-alcoholic fatty liver disease (NAFLD), which is seen in more than of 80% of patients with obesity, is becoming the most common cause of liver dysfunction.^3^

Bariatric surgery is considered not only to form a treatment for obesity, but also to be a means for improving related illnesses.^4^ Roux-en-Y gastric bypass (RYGB) is considered to be the gold standard for bariatric surgery.^5^ However, sleeve gastrectomy (SG) and one-anastomosis gastric bypass-mini-gastric bypass (OAGB-MGB) surgeries have challenged RYGB recently.^6^^,^^7^

Nonetheless, despite successful results from treating obesity and related complications using these techniques, there are concerns surrounding their restrictive and/or malabsorptive outcomes, which may be associated with long-term adverse consequences.^8^^,^^9^^,^^10^ These include protein malnutrition, manifested as albumin (Alb) levels of less than 3.5 g/dl, which may be associated with death, myocardial infarction and sepsis.^11^^,^^12^^,^^13^ In a number of previous studies, hypoalbuminemia after OAGB-MGB, RYGB and SG was reported.^14^^,^^15^^,^^16^ The current study was conducted in order to improve previous research data. Furthermore, no similar domestic study had compared the three types of surgery regarding Alb levels.

Liver failure is another complication after rapid weight loss post-bariatric surgery that was previously reported.^17^ Moreover, decreased liver transaminase levels were observed in other studies.^18^

## OBJECTIVE

Because of these contradictory findings and the lack of similar studies in Iran, the aim of this study was to investigate and compare changes in serum Alb and liver enzyme levels following three types of bariatric surgery. A secondary aim was to measure and compare body composition changes between groups.

## METHODS

### Patients and study design

This retrospective cohort study was conducted among 151 laparoscopic OAGB-MGB, SG and RYGB surgery patients within the past six months, among those referred to the obesity clinic of Hazrat-e Rasool General Hospital, Tehran, Iran (which is a Center of Excellence of the European Branch of the International Federation for Surgery of Obesity), between April 2018 and June 2019. Patients were enrolled in this study if they were aged 18-65 years and had preoperative body mass index (BMI) ≥ 40 kg/m^2^ or BMI ≥ 35 kg/m^2^ with major comorbidities such as T2DM, hypertension, CVD or dyslipidemia. Patients with a history of abdominal surgery and pregnancy after obesity surgery were excluded. Data on these patients relating to their condition pre-surgery and three and six months’ post-surgery were obtained from the National Iranian Obesity Surgery Database, which is the largest such database in Iran. This study was approved by the Health Ethics Committee of the Research Council of Tehran University of Medical Sciences (Ethics number: IR.TUMS.VCR.REC.1397.308; on July 23, 2018). A written informed consent form was received from all patients.

### Data collection

#### Basic information

Demographic information (age, sex, education and marital status), anthropometric indices (weight, height, BMI, waist and hip) and comorbidities (dyslipidemia, diabetes, hypertension and cardiovascular disease) were collected and recorded in the database by a qualified specialist. Height was measured, without shoes, to the nearest 0.5 cm using a Seca stadiometer (Seca 700, Hamburg, Germany). Body weight was measured with the patient wearing light clothing and no shoes, using a Seca scale (Seca 700, Hamburg, Germany). BMI and percentage total weight loss (%TWL) were calculated using the following formulas, respectively: BMI = weight (kg)/height^2^ (m); and $\%TWL\;=\;\left[\left(initial\;weight)\;-\;(postoperative\;weight\right)\right]/\left[\left(initial\;weight\right)\right]\;*\;100$. Waist and hip circumferences were measured using a nonelastic measuring tape, without imposing any pressure on the individual’s body, at the top of the iliac crest and at the largest part of the buttocks, respectively, to a precision of 0.1 cm.

#### Biochemical measurements

Fasting blood samples were taken to measure serum levels of albumin (Alb), alanine aminotransferase (ALT), aspartate aminotransferase (AST), alkaline phosphatase (ALKP), hemoglobin (Hb), hematocrit (Hct) and platelets (PLT). All measurements were made using standard laboratory methods.

#### Body composition measurements

A body composition analyzer (Tanita BC-418, Tanita Corp., Tokyo, Japan) was used to estimate fat range (%), fat mass (kg), fat-free mass (kg), muscle mass (kg), visceral fat (level) and body water (kg and %).

#### Dietary intake and physical activity assessment

A 24-hour dietary recall questionnaire (two workdays and one weekend day) was used to assess the dietary intake of each patient^19^ in their pre and postoperative states. Physical activity (PA) was assessed preoperatively and also six months postoperatively, by means of the International Physical Activity Questionnaire (IPAQ).^20^

#### Surgical technique

In SG surgery, about 80% of the stomach is removed from 3-5 cm of the pylorus. For gastric resection, a linear stapler is applied alongside a 36 (Fr) calibrating bougie to achieve a gastric volume of 50-100 cm^3^. The OAGB-MGB procedure technique has previously been reported.^21^ In the RYGB procedure, a small gastric pouch with a volume of 30-60 cm^3^ is created and connected to the Roux limb, with a length of 75-100 cm. The length of the biliopancreatic limb varies between 75 and 100 cm.

### Statistical analysis

The sample size of 144 individuals (48 per group) was estimated by considering a two-sided α = 0.05 and 80% power (β = 0.2). Due to the possibility of dropouts, the sample size was then increased by about 10%. Thus, 158 patients were enrolled by means of convenience sampling.

Data analysis was performed using the Statistical Package for the Social Sciences (SPSS) software, version 22.0 (IBM Corp., Armonk, New York, United States). Descriptive statistics were presented as mean ± standard deviation, or as frequencies and percentages. Repeated-measurement analysis was used to assess dependent variable changes over time (at the times of 0, 3 and 6 months) within each of the groups (SG, RYGB and OAGB-MGB). The differences in means relating to 0-3 and 0-6 months were compared between the groups by means of a univariate general linear model (GLM).

Comparison and analysis of patients’ dietary intake and their physical activity levels at two separate times (baseline and six months post-surgery) between the groups was performed using an analysis of variance (ANOVA) test; the paired-sample t test was also used in intragroup analyses on dietary intake and physical activity levels. P-values < 0.05 were considered statistically significant.

## RESULTS

**Figure 1.**
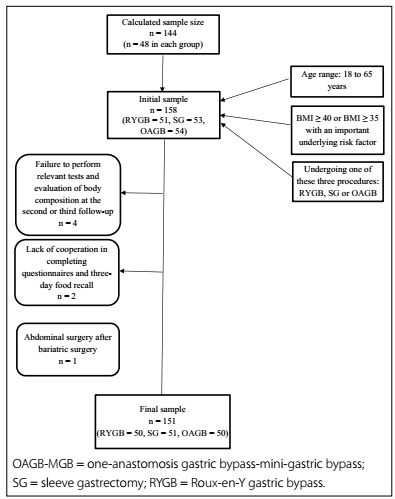
Flow chart of sample size.

Over the period from April 2018 to June 2019, among 158 eligible patients, 151 were included in the study (50 in the OAGB-MGB and RYGB groups, and 51 in the SG group) (Figure 1).

### Basic patient characteristics

In the study population, there were almost five times as many females as males (84.1% versus 15.9%). There was no difference among the three groups in terms of female or male gender (P = 0.369). The mean age was highest among the RYGB patients (43.04 ± 8.31 years) (P = 0.017). Also, the patients in the three groups were significantly different in terms of their educational levels (P < 0.001) and marital status (P = 0.003). At six months post-surgery, none of the participants reported smoking or alcohol consumption. Significantly, the number of participants with diabetes in the OAGB-MGB group was about four and three times higher than the SG and RYGB groups, respectively (P = 0.004). No statistical difference in any other comorbidities was found between the groups. The descriptive patient characteristics are shown in Table 1.

**Table 1.** Basic descriptive characteristics of the patients

Characteristics		OAGB-MGB (n = 50)	SG (n = 51)	RYGB (n = 50)	P-value
Age, years (SD)		39.54 (9.29)	37.73 (10.42)	43.04 (8.31)	0.017^*^
Gender, n (%)	Female	41 (82)	41 (80.4)	45 (90)	0.37
	Male	9 (18)	10 (19.6)	5 (10)	
Education, n (%)	Illiterate	0 (0)	1 (2)	0 (0)	0.000^*^
	1-6 years of education	4 (8)	15 (29.4)	12 (24)	
	7-12 years of education	31 (62)	13 (25.5)	31 (62)	
	12+ years of education	15 (30)	22 (43.1)	7 (14)	
Marital status, n (%)	Married	38 (76)	30 (58.8)	39 (78)	0.003^*^
	Single	8 (16)	18 (35.3)	3 (6)	
	Divorced	4 (8)	3 (5.9)	8 (16)	
Alcohol consumption^$^ n (%)	Yes, n (%)	3 (6)	9 (17.6)	3 (6)	0.08
	No, n (%)	47 (94)	42 (82.4)	47 (94)	
Smoking^§^, n (%)	Yes, n (%)	4 (8)	8 (15.7)	3 (6)	0.21
	No, n (%)	46 (92)	42 (82.4)	47 (94)	
Comorbidities, n (%)	Diabetes	15 (30)	4 (7.8)	5 (10)	0.004^*^
	Dyslipidemia	11 (22)	9 (17.6)	11 (22)	0.90
	HTN	8 (16)	8 (15.7)	12 (24)	0.48
	CVD	1 (2)	0 (0)	2 (4)	0.35

OAGB-MGB = one-anastomosis gastric bypass-mini gastric bypass; SG = sleeve gastrectomy; RYGB = Roux-en-Y gastric bypass; SD = standard deviation; BMI = body mass index; HTN = hypertension; CVD = cardiovascular diseases.

^§^Those who smoked tobacco in the past 30 days. ^$^Those who consumed alcohol in the past 30 days.

^*^P-value less than 0.05 was considered statistically significant.

### Physical activity and dietary intake

Analysis on physical activity revealed that all the patients had light activity (less than 600 metabolic equivalent (MET)-minutes/week) before surgery. However, six months post-surgery, the majority of the patients (96%) had moderate activity: 600-1500 MET-minutes/week.

Detailed information on the mean intake of energy and macronutrients is presented in Table 2. The total energy and macronutrient intakes at six months post-surgery were significantly lower than before surgery (P < 0.05). A one-way ANOVA test showed that there were no significant differences in terms of energy and macronutrient intakes among the SG, OAGB-MGB and RYGB groups at baseline (P > 0.05), but that significant differences existed between the three groups six months after surgery (Table 2). The Tukey post-hoc test revealed that energy and carbohydrate, protein and fat intakes were significantly higher in the OAGB-MGB group than in the SG group six months after surgery, with significance levels of 0.002, 0.010, 0.044 and 0.010, respectively.

**Table 2.** Nutrient composition of diets before and six months after three types of surgeries

	OAGB-MGB		SG		RYGB		P-value^*^	
	Preoperative	6 months postoperative	Pre-operative	6 months postoperative	Preoperative	6 months postoperative	Preoperative	6 months postoperative
Energy (kcal)	2036.25 ± 868.82	719.57 ± 239.31	2173.58 ± 620.89	525.57 ± 308.37	2162 ± 275.54	594.89 ± 274.71	0.51	0.003
Carbohydrate (g)	292.67 ± 138.39	88.95 ± 32.58)	301.46 ± 78.19	65.36 ± 46.96	315.88 ± 56.04	74.41 ± 36.26	0.51	0.013
Fat (g)	63.26 ± 35.38	24.20 ± 13.67	75.09 ± 30.04	17.07 ± 11.56	69.97 ± 17.00	18.97 ± 10.14	0.13	0.011
Protein (g)	77.92 ± 31.51	39.23 ± 15.68	81.99 ± 46.87	31.14 ± 18.17	77.25 ± 23.41	34.98 ± 15.47	0.78	0.057

Data expressed as mean ± SD. ^*^P-value result from ANOVA. P-values less than 0.05 were considered statistically significant.

OAGB-MGB = one-anastomosis gastric bypass-mini gastric bypass; SG = sleeve gastrectomy; RYGB = Roux-en-Y gastric bypass.

### Anthropometric indices, body composition and biochemical parameters

At the three-month follow-up, the mean percentage total weight loss (%TWL) after OAGB-MGB, SG and RYGB was 17.47% ± 4.39%, 18% ± 73% ± 4.55% and 19.56% ± 5.15%, respectively. The OAGB-MGB, SG and RYGB groups lost an average of 26.32% ± 5.13%, 26.59% ± 6.43% and 27.71% ± 4.64% of their total weight by the time of six months post-surgery, respectively. The %TWL differences at three and six months among the three groups were not statistically significant (P = 0.085 at three months and P = 0.805 at six months). The trends of changes in biochemical measurements, anthropometric and body composition, at different time points in the three surgery groups are reported in Table 3. According to the one-way repeated-measurement ANOVA results, the serum ALT levels were reduced significantly (P < 0.001) in all three groups. Additionally, significant changes in AST were noted (P = 0.003). However, the Alb and ALKP concentrations did not change significantly within the groups at various time points (P = 0.413 and P = 0.053, respectively). Moreover, the anthropometric parameters (weight, BMI, waist and hip) and body composition parameters (fat stores, muscle mass and fat-free mass) significantly decreased (P < 0.001) over the six-month period following surgery.

**Table 3.** Trends of serum and physical measurements at various time points in three types of surgical procedure

	OAGB-MGB			SG			RYGB			P-value^*^
	Preoperative	3 months postoperative	6 months postoperative	Preoperative	3 months postoperative	6 months postoperative	Preoperative	3 months postoperative	6 months postoperative	
Weight (kg)	120.1 ± 22.34	98.85 ± 17.57	88.26 ± 16.16	121.6 ± 18.36	98.51 ± 13.5	89 ± 13.87	118.26 ± 17.38	94.93 ± 13.87	86.10 ± 12.48	< 0.001
BMI (kg/m^2^)	45.91 ± 6.95	39.06 ± 10.11	33.77 ± 5.30	45.23 ± 5.97	36.69 ± 4.67	33.14 ± 4.83	45.01 ± 4.91	36.16 ± 4.23	32.81 ± 3.95	< 0.001
Waist (cm)	117.52 ± 13.62	100.91 ± 11.28	93.23 ± 10.01	115.93 ± 9.58	97.43 ± 8.52	93.81 ± 8.27	116.64 ± 9.86	98.23 ± 7.87	91.79 ± 8.43	< 0.001
Hip (cm)	134.17 ± 11.61	118.94 ± 11.26	111.44 ± 9.18	135.62 ± 7.98	119.65 ± 7.62	113.81 ± 9.15	136.76 ± 8.99	120.86 ± 9.02	112.14 ± 9.25	< 0.001
Fat range (%)	45.19 ± 8.64	40.68 ± 6.94	35.05 ± 8.09	46.79 ± 5.51	40.69 ± 6.11	36.48 ± 6.99	47.58 ± 4.59	40.59 ± 5.96	35.38 ± 6.99	< 0.001
Fat mass (kg)	55.01 ± 14.01	40.12 ± 11.13	31.15 ± 10.34	56.71 ± 12.31	40.21 ± 10.62	32.76 ± 11.72	56.59 ± 11.13	38.69 ± 81.88	30.45 ± 8.59	< 0.001
Fat free mass (kg)	64.21 ± 13.94	58.13 ± 11.74	56.65 ± 11.13	64.07 ± 9.80	57.77 ± 7.77	55.72 ± 7.61	62.04 ± 11.05	56.25 ± 9.14	54.94 ± 8.64	< 0.001
Visceral fat (level)	16.46 ± 5.68	11.78 ± 3.75	8.98 ± 3.41	15.96 ± 4.03	11.09 ± 3.14	8.78 ± 3.10	15.90 ± 4.13	10.72 ± 2.61	8.40 ± 2.59	< 0.001
Muscle mass (kg)	60.97 ± 13.33	55.16 ± 11.30	53.79 ± 10.68	60.83 ± 9.41	54.88 ± 7.47	52.93 ± 7.33	58.87 ± 10.55	53.39 ± 8.73	52.17 ± 8.29	< 0.001
Body water (%)	47.00 ± 10.20	42.50 ± 8.62	41.47 ± 8.15	46.88 ± 7.24	42.25 ± 5.74	40.73 ± 5.62	45.41 ± 8.09	41.19 ± 6.69	40.23 ± 6.32	< 0.001
Body water (kg)	39.53 ± 4.24	43.41 ± 5.08	47.62 ± 6.02	39.04 ± 4.01	43.48 ± 4.49	46.55 ± 5.14	38.37 ± 3.36	43.71 ± 4.42	47.53 ± 5.17	< 0.001
Hb (mg/dl)	13.44 ± 1.54	13.38 ± 1.37	13.12 ± 1.41	13.72 ± 1.41	13.98 ± 1.31	13.89 ± 1.40	13.45 ± 1.31	13.32 ± 1.41	13.03 ± 1.39	0.015
HCT (%)	40.79 ± 4.22	40.21 ± 5.66	39.49 ± 3.65	41.51 ± 3.59	43.08 ± 6.72	41.62 ± 3.83	40.79 ± 3.25	40.85 ± 9.20	39.61 ± 3.94	0.091
PLT (10^3^/mm^3^)	276.96 ± 69.88	244.63 ± 78.97	257.41 ± 71.13	297.57 ± 65.21	255.65 ± 65.14	266.35 ± 72.79	299.49 ± 78.57	256.33 ± 69.35	266.49 ± 67.74	< 0.001
Alb (g/l)	4.27 ± 0.36	4.32 ± 0.52	4.21 ± 0.37	4.42 ± 0.40	4.37 ± 0.41	4.35 ± 0.38	4.31 ± 0.39	4.28 ± 0.33	4.28 ± 0.51	0.41
SGOT (U/l)	18.56 ± 6.51	25.86 ± 13.61	20.23 ± 8.05	27.40 ± 22.90	21.99 ± 9.38	18.56 ± 5.42	22.11 ± 15.25	22.86 ± 12.16	19.05 ± 5.70	0.003
SGPT (U/l)	23.38 ± 3.51	28.45 ± 16.36	19.65 ± 10.58	34.22 ± 27.97	25.24 ± 16.37	17.70 ± 7.86	26.30 ± 17.22	25.55 ± 17.01	18.85 ± 7.82	< 0.001
ALKP (U/l)	187.60 ± 54.99	184.00 ± 45.53	198.48 ± 47.43	170.28 ± 59.76	153.16 ± 50.10	155.35 ± 56.23	191.38 ± 60.32	182.09 ± 49.04	193.20 ± 54.35	0.053

OAGB-MGB = one-anastomosis gastric bypass-mini gastric bypass; SG = sleeve gastrectomy; RYGB = Roux-en-Y gastric bypass; BMI = body mass index; Hb = hemoglobin; Hct = hematocrit; SGOT = serum glutamic oxaloacetic transaminase; SGPT = serum glutamic-pyruvic transaminase; ALKP = alkaline phosphatase; BUN = blood urea nitrogen; Alb = albumin.

Data are expressed as mean ± standard deviation. ^*^P-values are results from repeated-measurement one-way analysis of variance. P-values less than 0.05 were considered statistically significant.

### Influence of OAGB-MGB, SG and RYGB on physical and blood parameters

The differences in the means of the anthropometric, body composition and biochemical parameters at three and six months after the three different types of bariatric surgery are presented in Table 4. Using univariate analysis from the general linear model (GLM), the effects of confounding variables were controlled for, including age, gender, education level, marital status, alcohol consumption and diabetes, as dependent variables.

**Table 4.** Results from univariate general linear model (GLM) for the mean differences in variables at three and six months after surgery

		OAGB-MGB (n = 50)	SG (n = 51)	RYGB (n = 50)	P-value
Diff-Weight (kg)	0-3	-21.25 ± 7.56	-23.09 ± 7.59	-23.33 ± 8.11	0.13
	0-6	-31.84 ± 9.44	-32.60 ± 9.93	-32.16 ± 8.46	0.46
Diff-Waist (cm)	0-3	-16.10 ± 8.07	-15.72 ± 5.87	-18.37 ± 8.40	0.096
	0-6	-26.76 ± 14.14	-23.41 ± 10.60	-24.35 ± 7.77	0.52
Diff-Hip (cm)	0-3	-15.77 ± 7.80	-15.99 ± 8.10	-16.27 ± 7.70	0.84
	0-6	-23.60 ± 7.22	-24.16 ± 11.48	-24.20 ± 8.85	0.81
Diff-BMI (kg/m^2^)	0-3	-6.86 ± 9.69	-8.54 ± 2.59	-8.85 ± 2.77	0.096
	0-6	-12.14 ± 3.15	-12.08 ± 3.44	-12.19 ± 2.69	0.68
Diff-Fat range (%)	0-3	-4.51 ± 7.13	-6.10 ± 2.77	-7.00 ± 3.63	0.01^b^
	0-6	-10.14 ± 8.42	-10.31 ± 4.04	-12.21 ± 4.40	0.04^b^
Diff-FM (kg)	0-3	-14.89 ± 6.59	-16.61 ± 5.32	-17.90 ± 8.02	0.035^b^
	0-6	-23.86 ± 8.48	-23.95 ± 11.73	-26.14 ± 8.89	0.28
Diff- FFM (kg)	0-3	-6.08 ± 3.48	-6.30 ± 3.32	-5.79 ± 3.33	0.91
	0-6	-7.55 ± 4.32	-8.35 ± 4.10	-7.11 ± 3.67	0.86
Diff-MM (kg)	0-3	-5.80 ± 3.23	-5.95 ± 3.16	-5.48 ± 3.17	0.93
	0-6	-7.18 ± 4.02	-7.90 ± 3.88	-6.70 ± 3.47	0.87
Diff-VF	0-3	-4.68 ± 2.77	-4.86 ± 2.01	-5.18 ± 3.03	0.19
	0-6	-7.48 ± 3.73	-7.18 ± 2.70	-7. 50 ± 3.45	0.24
Diff-TBW (%)	0-3	-4.50 ± 2.51	-4.61 ± 2.43	-4.22 ± 2.94	0.10
	0-6	-5.53 ± 3.16	-6.15 ± 3.01	-5.18 ± 3.08	0.84
diff-BW (kg)	0-3	3.88 ± 2.29	4.47 ± 2.04	5.34 ± 2.90	0.006^b^
	0-6	8.09 ± 3.86	7.51 ± 2.97	9.16 ± 3.48	0.025^a^
diff-Alb (g/l)	0-3	0.05 ± 0.51	-0.04 ± 0.53	-0.03 ± 0.47	0.40
	0-6	-0.07 ± 0.54	-0.06 ± 0.52	-0.03 ± 0.57	0.70
diff-SGOT (U/l)	0-3	7.30 ± 13.09	-5.41 ± 18.36	0.75 ± 17.77	0.005^c^
	0-6	1.67 ± 8.70	-8.69 ± 21.91	-3.06 ± 16.99	0.02^c^
diff-SGPT (U/l)	0-3	5.07 ± 15.15	-8.98 ± 22.31	-0.76 ± 20.43	0.006^c^
	0-6	-3.73 ± 13.14	-16.23 ± 26.94	-7.45 ± 18.00	0.017^c^
diff-ALKP (U/l)	0-3	-3.40 ± 51.54	-18.08 ± 57.42	-9.29 ±51.04	0.16
	0-6	11.32 ± 52.87	-13.91 ± 56.47	3.70 ± 60.10	0.041^c^

Data are expressed as mean ± standard deviation. OAGB-MGB = one-anastomosis gastric bypass-mini-gastric bypass; SG = sleeve gastrectomy; RYGB = Roux-en-Y gastric bypass; Diff = mean difference; (0-3) = mean difference in variables three months after surgery, compared with preoperative time; (0-6) = mean difference in variables six months after surgery, compared with preoperative time; BMI = body mass index; BMR = basal metabolic rate; FM = fat mass; FFM = fat-free mass; MM = muscle mass; VF = visceral fat level; TBW = total body water; BW = body water; SGOT = serum glutamic-oxaloacetic transaminase; SGPT = serum glutamic-pyruvic transaminase; Alb = albumin; ALKP = alkaline phosphatase. P-values are results from univariate GLM and are significant at the 0.05 level. ^a,b,c^Pairwise comparison adjustment for multiple comparisons: Bonferroni; ^b^Significant difference between OAGB-MGB and RYGB; ^a^Significant difference between SG and RYGB; ^c^Significant difference between SG and OAGB-MGB.

Regarding mean differences in dependent variables and operations as fixed factors in the univariate model, there were no significant differences in albumin levels among the groups post-surgery (P > 0.05). Despite the postoperative fluctuations in the serum levels of liver enzymes (AST, ALT and ALKP) that were observed in both the OAGB-MGB and the RYGB group, all of these enzymes in the SG group showed significant decreasing trends during the study. Interestingly, SG was significantly more effective than OAGB-MGB in lowering AST (P = 0.003 at three months and P = 0.015 at six months), ALT (P = 0.005 at three months and P = 0.015 at six months) and ALKP (P = 0.037 at six months). Additionally, significant differences were found among the three groups in terms of the fat range percentage at both times (P = 0.014 and P = 0.036 at three and six months, respectively). Interestingly, RYGB, in comparison with OAGB-MGB, had a greater effect on fat range reduction (P = 0.011 at three months; P = 0.059 at six months). Moreover, fat mass reduction at three months post-surgery was highest in RYGB patients, and there was a considerable difference with OAGB-MGB in pairwise comparisons (P = 0.042).

## DISCUSSION

This study focused on changes in serum Alb levels and liver enzymes in 151 patients who had undergone RYGB, OAGB-MGB or SG. Significant changes in serum AST and ALT levels were noted during the follow-up. Additionally, in the intergroup comparison, SG showed a significant effect towards reducing both transaminases at both time points, and on ALKP levels at six months, compared with OAGB-MGB. Changes in serum Alb levels were not significantly different among the three groups.

Weight loss-induced improvements in liver enzyme levels among patients receiving bariatric surgery (non-adjustable or adjustable banding, vertical banded gastroplasty or gastric bypass) have previously been reported.^18^ Bariatric surgery reduces transaminase levels^22^ by reducing liver fat and inflammation, and also by improving insulin resistance following appetite loss and calorie restriction.^23^ Despite the reported importance of weight loss in relation to reduction of liver enzymes, the present study revealed that SG played a vital role in reducing liver enzymes compared with the two other surgical methods, especially OAGB-MGB; a downward trend of weight was observed in all three groups, with no statistically significant differences. The presence of a lower number of patients with diabetes in the SG group (7.8%) than in the RYGB group (10%), and particularly lower than in the OAGB-MGB group (30%), might explain this finding. It is worth noting that, in the clinic of the present study, the main reason given for performing a higher proportion of OAGB-MGB surgeries among patients with diabetes was its greater effectiveness in lowering blood sugar, compared with other types of surgery.^24^^,^^25^ This is supported by a previous study that indicated that SG resulted in greater liver enzyme improvement, compared with RYGB.^26^ However, no data comparing SG and OAGB-MGB were available. Several mechanisms have been proposed for explaining the increased levels of liver enzymes after OAGB-MGB:

1. Worse fatty liver levels following OAGB-MGB, with increased levels of liver enzymes.^27^ In this regard, diagnostic evaluation of hepatic steatosis seems to be an important factor. However, in the clinic of the present study, no routine evaluation of liver steatosis and fibrosis was performed within the short-term postoperative assessments, which was in accordance with the guidelines of the American Society for Metabolic and Bariatric Surgery (ASMBS)^28^ (0, 3 and 6 months).
2. Liver enzyme levels can also be increased through growth of intestinal bacterial flora, which leads to production of hepatotoxic macromolecules that are transported to the liver through the portal vein. In vulnerable livers facing nutritional challenges, this can lead to liver damage.^29^
3. Malabsorption and malnutrition after OAGB-MGB seem to be an underlying mechanism involved in increased liver enzyme levels.^30^ In this regard, we assessed the patients’ dietary intake, although we did not study the link between liver enzymes and markers of malnutrition, except albumin.

In this context, no significant changes in serum Alb levels were noted in any group of the present study. Additionally, the intergroup comparison of mean difference of Alb at three and six months after surgery did not show any statistically significant difference. This result was in agreement with some previous findings.^31^^,^^32^ However, Jammu and Sharma found that the prevalence of hypoalbuminemia was lower in a SG group and higher in an OAGB-MGB group. Those authors suggested that being vegetarian, having diabetic nephropathy, having alcoholic or nonalcoholic fatty liver disease and presenting long bypass limb length were possible causes of albumin deficiency. However, their long-term follow-up (maximum 87 months and minimum 20 months), in comparison with the short follow-up of the present study (six months) may explain this discrepancy. Likewise, the 24-hour dietary recall analysis of the present study showed that energy and protein intake in malabsorptive surgery groups (especially OAGB-MGB) were higher than in the SG group. No exact measurement of dietary protein intake was made in Jammu and Sharma’s study: their patients were only recorded as having a high-protein diet based on self-reported statements.

Significant reductions in anthropometric and body composition parameters were observed in all three groups. The greatest reduction in body fat range and fat mass at six months post-surgery was observed in the RYGB group. This reduction was significant, compared with the OAGB-MGB group.

Weight loss and decreases in BMI and waist, and hip circumference have previously been reported after bariatric surgery.^33^ However, the downward trend of anthropometric indices was not significantly different among the three groups. Additionally, there was no statistical difference in %TWL between three groups. This result was also consistent with previous findings, which showed that SG may be correlated with malabsorptive bariatric surgery aimed at weight loss.^34^ However, some inconsistencies have also been observed.^35^ The main reason for the same %TWL and BMI loss between the three groups seems to have been patient-tailored surgery, as decided by the surgeon.

Loss of body fat reserves, along with fat-free mass and muscle mass wasting, was found in all groups, which concurs with similar post-bariatric surgery studies.^36^ This may relate to significant restrictions of energy and macronutrient intake (Table 2). Additionally, the fat range percentage and fat mass in the RYGB group were lower than in the other two groups, especially the OAGB-MGB group, while the reductions in fat-free mass and muscle tissue after the three types of surgery did not differ significantly. This was contrary to the findings of Arble et al. (2018),^37^ who showed that both RYGB and OAGB-MGB surgeries had positive effects on fat reduction, compared with SG, and that there was no significant difference in body fat loss between RYGB and OAGB-MGB. Also, no change in muscle tissue was observed by Arble et al. after surgery, compared with a control group. These inconsistencies may have been due to differences in study designs (human versus animal study).

Additionally, the role of physical activity in maintaining muscle mass cannot be ignored.^38^^,^^39^ Physical activity in all study groups improved from mild to moderate after surgery. However, concurrent food intake reductions were also observed in all groups. The OAGB-MGB group had higher energy and macronutrient consumption than the other groups, while the weight change among the groups was statistically similar. This suggests that OAGB-MGB patients may experience greater decreases in nutrient absorption than RYGB and SG patients. A rat study also showed that the OAGB-MGB group was more malabsorbent, showing greater protein and calorie excretion than the RYGB group. Likewise, slightly higher food intake in the OAGB-MGB group was observed, which was attributed to the increased expression of orexigenic peptides (neuropeptide Y and N-acetyl-γ-glutamyl-phosphate) in the rat hypothalamus.^40^

One of the strengths of the present study was that changes in liver enzymes, serum Alb, body composition parameters, dietary intake and physical activity were simultaneously evaluated in three surgical groups. However, the sample selection from a single obesity clinic in Hazrat-e Rasool General Hospital may have been a limitation (nonetheless, patients are referred nationally, and thus the results may be generalized with minimum bias). Furthermore, the lack of dietary measurement and physical activity at three months post-surgery was another limitation. Additionally, due to the retrospective nature of the data sources, we could not control for some specific variables, such as the preoperative severity of steatosis and steatohepatitis, by either direct or indirect means.

## CONCLUSION

The findings from this study provide support regarding the ability of SG to reduce the serum levels of AST, ALT and ALKP; and the ability of RYGB to reduce body fat, compared with OAGB-MGB surgery, within short-term follow-up. However, all of these types of surgery were found to be equally effective regarding serum albumin changes and %TWL at the six-month follow-up. This study may lead to greater insights into the various surgical procedures for patients with different blood parameters and body composition conditions.
